# Hospitalization for epistaxis: a population-based healthcare research study in Thuringia, Germany

**DOI:** 10.1007/s00405-020-05875-2

**Published:** 2020-03-02

**Authors:** Max Kallenbach, Andreas Dittberner, Daniel Boeger, Jens Buentzel, Holger Kaftan, Kerstin Hoffmann, Peter Jecker, Andreas Mueller, Gerald Radtke, Orlando Guntinas-Lichius

**Affiliations:** 1 Department of Otorhinolaryngology, Jena University Hospital, Am Klinikum 1, 07740 Jena, Germany; 2 Department of Otorhinolaryngology, Zentralklinikum, Suhl, Germany; 3 Department of Otorhinolaryngology, Südharz-Krankenhaus gGmbH, Nordhausen, Germany; 4 Department of Otorhinolaryngology, Helios-Klinikum, Erfurt, Germany; 5 Department of Otorhinolaryngology, Sophien/Hufeland-Klinikum, Weimar, Germany; 6 Department of Otorhinolaryngology, Klinikum Bad Salzungen, Bad Salzungen, Germany; 7 Department of Otorhinolaryngology, SRH Wald-Klinikum, Gera, Germany; 8 Department of Otorhinolaryngology, Ilm-Kreis-Kliniken, Arnstadt, Germany

**Keywords:** Bleeding, Epistaxis, Hospitalization, Diagnostics, Treatment, Nasal packing, Healthcare research, Anticoagulation

## Abstract

**Purpose:**

Epistaxis is the most common ENT emergency. The aim was to determine population-based data on severe epistaxis needing inpatient treatment.

**Methods:**

Retrospective population-based cohort study in the federal state Thuringia in 2016 performed on all 840 inpatients treated for epistaxis in otolaryngology departments (60.1% male, median age: 73 years; 63.9% under anticoagulation). The association between patients’ and treatment characteristics and longer inpatient stay (≥ 4 days) as well as readmission for recurrent epistaxis was analyzed using univariable and multivariable statistics.

**Results:**

The overall incidence of epistaxis needing inpatient treatment was higher for men (42 per 100,000) than for women (28 per 100,000). The highest incidence was reached for men > 85 years (222 per 100,000). Most important independent predictors for longer inpatient stay were localization of the bleeding not in the anterior nose (OR = 2.045; CI = 1.534–2.726), recurrent bleeding during inpatient treatment (OR = 2.142; CI = 1.508–3.042), no electrocoagulation (OR = 2.810; CI = 2.047–3.858), and blood transfusion (OR = 2.731; CI = 1.324–5.635). Independent predictors for later readmission because of recurrent epistaxis were male gender (OR = 1.756; CI = 1.155–2.668), oral anticoagulant use (OR = 1.731; CI = 1.046–2.865), and hereditary hemorrhagic telangiectasia (OR = 13.216; CI 5.102–34.231).

**Conclusions:**

Inpatient treatment of epistaxis seems to be variable in daily routine needing standardization by clinical guidelines and strategies to shorten inpatient treatment and to reduce the risk of readmission.

**Electronic supplementary material:**

The online version of this article (10.1007/s00405-020-05875-2) contains supplementary material, which is available to authorized users.

## Introduction

Epistaxis is a frequent emergency workload in ENT departments and many patients require an inpatient treatment [1–3]. As a result of the changing demographic structure, the number of older patients using classical anticoagulants like Vitamin K antagonists (VKA) is increasing [4, 5]. Furthermore, new oral non-VKA oral anticoagulants (NOAC) are increasingly prescribed. The increased use of classical and new anticoagulants seems to increase the incidence of epistaxis [5]. The influence on more severe epistaxis and recurrent bleeding needing inpatient treatment is less clear [3, 5]. The majority of data on epistaxis is based on hospital-based analyses. There is a lack of epidemiological, population-based data, especially for complicated recurrent epistaxis with readmission [3, 6–8]. Hence, not much is known about the burden for the general population, especially on the burden of severe epistaxis needing inpatient treatment.

Thuringia is a territorial state in Germany with approximately 2.2 Mio. inhabitants. There are only eight hospitals with departments of otolaryngology in Thuringia. The departments of otolaryngology have built a network primarily to improve health services research in the field of otolaryngology [9–13]. Use of this network provided an ideal platform for a population-based analysis of the inpatient treatment of epistaxis in daily practice in the year 2016 in Thuringia. The focus was on predictors for inpatient length of stay and for the risk of readmission because of recurrent epistaxis.

## Methods

### Study design and patients

The institutional ethics committee approved the study protocol for a retrospective data collection. A standardized retrospective analysis was performed in all eight Thuringian hospitals with a department of otolaryngology. All patients were selected who were coded for epistaxis (R04.0) due to the International Classification of Diseases [ICD], 10th revision, German modification; ICD-10-GM) and who were hospitalized for epistaxis in 2016 (index treatment). If a readmission for recurrent epistaxis occurred within 12 months, the readmission could occur in 2016 and for some cases also in 2017). A retrospective search of the patients’ charts was performed, and the following variables were obtained: age, sex, medication, comorbidity, physical examinations, all diagnostics, medical treatment, and surgical procedures related to the epistaxis. Before analysis, the data were blinded with respected to the treating hospital. A systolic blood pressure value of ≥ 140 mmHg and a diastolic value of ≥ 90 mmHg defined as hypertension at admission. A systolic value of ≥ 180 mmHg and/or a diastolic value of ≥ 120 mmHg defined a hypertensive crisis. Anterior nose bleeding was defined as bleeding in the cartilaginous part of the septum (level of area I/II) including the Kiesselbach plexus. A bleeding posterior to the plexus was defined as posterior epistaxis.

The epidemiological calculations were based on the annual mean number of habitants in Thuringia in 2016. Population numbers were used that were given in the online database of the Thuringian State Office for Statistics (www.tls.thueringen.de).

### Statistical analysis

If not indicated otherwise, data are presented with mean values ± standard deviation (SD). All statistical analyses were performed using IBM SPSS, version 25. Parameter with > 10 cases per item was included into further subanalysis. The Chi-square test was used to compare nominal data of two independent subgroups. The Mann–Whitney *U*-test was used to compare scaled data of two independent subgroups. Parameters from these univariable statistical tests with *p* < 0.1 were included into multivariable binary logistic regression models with stepwise entry to analyse independent associations. Nominal *p* values of two-tailed tests are reported. The significance level for the multivariable analyses was set at *p* < 0.05.

## Results

### Subjects, diagnostics, and treatment

During the study period of 1 year, 840 patients (60.1% male, median age: 73 years) were admitted for epistaxis in Thuringia. Table 1 shows the characteristics of the study cohort. The majority of the patients (63.9%) used anti-platelet medications or anticoagulants (Vitamin K antagonist [VKA] and/ or Non-VKA oral anticoagulant [NOAC]). 12.9% had a double or triple anticoagulation. Arterial hypertension (57.7%) was the most frequent comorbidity. Bleeding was localized in about half of the patients (54.9%) in the anterior nose. The source of bleeding was not localized in 12.7%. The number of cases was lowest in the summer time. The number of inpatient treatments in 2016 varied from 1 to 9. The median duration of the inpatient treatment was 4 days. Supplement Table S1 summarizes the blood values and blood pressure measurements at admission. 20% of the patients had an international normalized ratio (INR) greater than 2.0, and 3.6% greater than 3.5. Half of the patients were hypertensive (49.6%), and 12.7% had a hypertensive crisis. Table 2 gives an overview over the treatment. Nasal packing was the most frequent single measure used in 70.5% of the patients. Median nasal packing time was 1 day (range: 0–6). Electrocoagulation was used in 41.1% of the patients. Chemical etching, vessel ligation, or embolization were rarely used (2.5%, 0.7%, 0.4% of the patients, respectively). Overall, 41.8% needed surgery and in 17.4% in general anesthesia. Anticoagulant medication was stopped in 21% of the anticoagulant users. Furthermore, 17.5% needed a substitution of the anticoagulant (in most cases by enoxaparin).

**Table 1:** Characteristics of all patients with hospitalization because of epistaxis in 2016 (*N* = 840)

Parameter	*N*	%
*Gender*		
Male	505	60.1
Female	335	39.9
*Residence in Thuringia*		
Yes	759	90.4
No	81	9.6
*Month of presentation*		
January	88	10.5
February	73	8.7
March	102	12.1
April	77	9.2
May	78	9.3
June	52	6.2
July	62	7.4
August	44	5.2
September	47	5.6
October	67	8.0
November	67	8.0
December	83	9.9
*Weekend or holiday*		
Yes	243	28.9
No	597	71.1
*Localization of the bleeding*		
Anterior	461	54.9
Posterior	166	19.8
Anterior and posterior	9	1.1
Tumor*	6	0.7
Not locatable	107	12.7
Not documented	91	10.8
*Recurred episodes of active bleeding during same inpatient treatment*		
Yes	184	21.9
No	656	78.1
*Patient under anticoagulation/blood thinner*		
Yes	536	63.9
No	304	36.2
*Number of anticoagulant drugs*		
Single anticoagulation	428	51.0
Double anticoagulation	89	10.6
Triple anticoagulation	19	2.3
No anticoagulation	304	36.2
*Type of anticoagulant/blood thinner*		
Anti-platelet drug	280	33.3
Vitamin K antagonist (VKA)	182	21.7
Non-VKA oral anticoagulant (NOAC)	128	15.2
*Comorbidity, epistaxis-relevant*		
Hypertension, arterial	485	57.7
Diabetes mellitus	193	22.9
Malignant tumor	79	9.4
Coronary heart disease	70	8.3
Anemia	41	4.9
Hereditary hemorrhagic telangiectasia	21	2.5
History of septoplasty	10	1.2
Alcohol abuse	10	1.2
Idiopathic thrombocytopenic purpura	9	1.1
Nasal septum perforation	8	1.0
History of FESS	7	0.8
Pregnancy	5	0.6
Factor V Leiden disease	3	0.4
Willebrand–Jürgens syndrome	1	0.1
Bernard–Soulier syndrome	1	0.1
*Active bleeding at arrival*		
Yes	612	72.9
No	228	27.1
	Mean ± SD	Median, range
Age, years	66.6 ± 20.9	73, 1–99
Duration of inpatient treatment, days	4.2 ± 3.2	4.0, 1–49
Number of inpatient treatments in 2016	1.2 ± 0.6	1, 1–9
Number of outpatient/inpatient treatments in 2016	1.3 ± 1.1	1, 1–20
Number of anticoagulants in patients under anticoagulation	1.2 ± 0.5	1, 1–3

^*^Tumor diagnosed later during inpatient work-up

*FESS* functional endoscopic sinus surgery, *SD* standard deviation

**Table 2:** Overview about the used therapeutic methods

Parameter	*N*	%
*Cold package*		
Yes	214	25.5
No	626	75.7
*Nasal packing*		
Yes	592	70.5
No	248	29.5
*Pause anticoagulant*		
Yes	117	13.9
No	419	49.8
No anticoagulant treatment	304	36.2
*Substitution of the anticoagulant*		
Yes	94	11.2
No	442	52.6
No anticoagulant treatment	304	36.2
*Any kind of surgery*		
Yes	505	60.1
No	335	39.9
*Anesthesia*		
No	449	53.5
Local	245	29.2
General	146	17.4
*Electrocoagulation*		
Yes	345	41.1
No	495	58.9
*Cauterization with silver nitrate or trichloroacetic acid*		
Yes	21	2.5
No	819	97.5
*Embolization*		
Yes	3	0.4
No	837	99.6
*Vessel ligation*		
Yes	6	0.7
No	834	99.3
*Further analgesia*		
Yes	118	14.0
No	722	86.0
*Nasal oil, ointment*		
Yes	691	82.3
No	149	17.7
*Antibiotics*		
Yes	238	28.3
No	602	71.7
*Antihypertensive drugs*		
Yes	101	12.0
No	739	88.0
*Fluid replacement*		
Yes	130	15.5
No	710	84.5
*Blood transfusion*		
Yes	40	4.8
No	800	95.2
*Iron*		
Yes	23	2.7
No	817	97.3
*Vitamin K*		
Yes	27	3.2
No	813	96.8
*Coagulation factor substitution*		
Yes	5	0.6
No	835	99.4
*Thrombocyte substitution*		
Yes	2	0.2
No	838	99.8
*Transferal to intensive care unit*		
Yes	21	2.5
No	819	97.5
	Mean ± SD	Median, range
Number of surgeries per patient	0.6 ± 0.6	1.0, 0–5
Number of drug treatments per patient	1.9 ± 1.1	2.0, 0–6
Nasal packings per patient	1.1 ± 0.9	1.0, 0–7
Duration of nasal packing, days	1.1 ± 1.0	1.0, 0–6

*SD* standard deviation

### Incidence of inpatient treatment for epistaxis

**Fig. 1:**
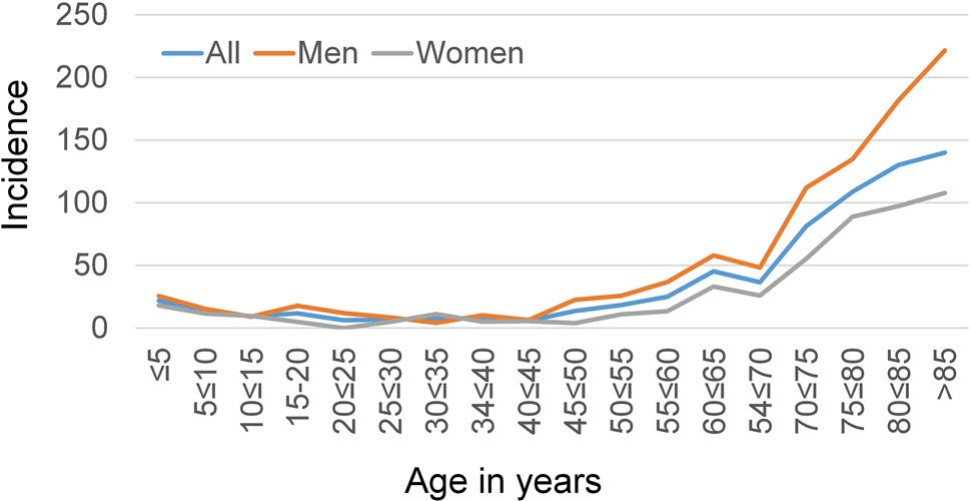
Incidence (N/100.000 population) of inpatient epistaxis treatment for the different age cohorts in Thuringia in 2016 of the patients living in Thuringia (*N* = 763; 90.8% of the study population)

Overall, the incidence for the need of an inpatient treatment for the patients living in Thuringia (90.8% of the study population) was 35 per 100,000 persons (Supplement Table 2). The incidence was higher for men (42 per 100,000) than for women (28 per 100,000). This gender difference became particularly evident for patients older than 70 years of age (Fig. 1). The incidence continuously increased for both sexes for patients older than 45 years with a steep increase for patients above 70 years. The highest incidence was reached for men older than 85 years with an incidence of 222 per 100,000 persons.

### Predictors for the duration of the inpatient treatment

Half of the patients (50.6%) had an inpatient length of stay of ≥ 4 days. Supplement Table 3 gives a summary over the univariate analyses on predictors for a longer length of inpatient stay (≥ 4 days). Anti-platelet drug use, anticoagulant combination therapy with 2–3 drugs, need for pause of the anticoagulant therapy, arterial hypertension, diabetes mellitus, localization of the bleeding not in the anterior nose, recurrent bleeding during the inpatient stay, need for nose packing, no electrocoagulation, and need for blood transfusion were univariate predictors for a longer inpatient stay (all *p* < 0.1). The multivariate analyses with these factors revealed several independent predictors for longer inpatient treatment (Table 3). The following patients’ characteristics were independent predictors (all *p* < 0.05): anti-platelet drug (Odds ratio [OR] = 1.825; 95% confidence interval [CI] = 1.221–2.729), need for pause of anticoagulation therapy (OR = 1. 856; CI = 1.295–2.659), arterial hypertension (OR = 1.415; CI = 1.052–1.902), diabetes mellitus (OR = 1.489; CI = 1.050–2.113), localization of the bleeding not in the anterior nose (OR = 2.045; CI = 1.534–2.726), and recurrent bleeding during inpatient treatment (OR = 2.142; CI = 1.508–3.042). Furthermore, the following treatment factors were predictive: nose packing (OR = 2.568; CI = 1.822–3.619), no electrocoagulation (OR = 2.810; CI = 2.047–3.858), and blood transfusion (OR = 2.731; CI = 1.324–5.635).

**Table 3:** Independent associations between patients’ characteristics, treatment, and the probability to have a shorter (1–3 days; reference) or longer (≥ 4 days) days of inpatient treatment

Measure		OR*	95% CI* lower	95% CI* upper	*p*
*Model 1: patients’ characteristics*					
Anti-platelet drug	No	1			
	Yes	1.825	1.221	2.729	**0.003**
Anticoagulant combination therapy, number of drugs	0 or 1 drug	1			
	2 or 3 drugs	1.223	0.659	2.268	0.523
Pause of anticoagulation	No	1			
	Yes	1.856	1.295	2.659	**0.001**
Hypertension, arterial	No	1			
	Yes	1.415	1.052	1.902	**0.022**
Diabetes mellitus	No	1			
	Yes	1.489	1.050	2.113	**0.026**
Localization of the bleeding	Anterior	1			
	Not anterior	2.045	1.534	2.726	**< 0.001**
Recurrent bleeding during inpatient treatment	No	1			
	Yes	2.142	1.508	3.042	**< 0.001**
*Model 2: treatment*					
Nose packing	No	1			
	Yes	2.568	1.822	3.619	**< 0.001**
Electrocoagulation	Yes	1			
	No	2.810	2.047	3.858	**< 0.001**
Blood transfusion	No	1			
	Yes	2.731	1.324	5.635	**0.007**

Multivariable binary logistic regression for the dichotomized outcome parameter length of inpatient stay: OR related to a shorter length of stay. Significant *p* values (*p* < 0.05) in bold

*OR* odds ratio, *CI* confidence interval

### Predictors for readmission for recurrent epistaxis

One hundred and thirty-four (134) patients (16.0%) were readmitted within 12 months for recurrent epistaxis. The results of the univariate analyses on predictors for readmission because of recurrent epistaxis are listed in Supplement Table 4. Male gender, use of non-vitamin K antagonist (VKA) oral anticoagulants (NOAC), use of 2–3 anticoagulants, length of index admission ≥ 4 days, hereditary hemorrhagic telangiectasia, recurrent bleeding during inpatient treatment, and nose packing were univariate predictors for readmission (all *p* < 0.1). The multivariate analyses with these factors revealed several independent predictors (all *p* < 0.05) for later readmission (Table 4). The following patients’ characteristics were independent predictors: male gender (OR = 1.756; CI = 1.155–2.668), NOAC use (OR = 1.731; CI = 1.046–2.865), hereditary hemorrhagic telangiectasia (OR = 13.216; CI 5.102–34.231), and recurrent bleeding during index inpatient treatment (OR = 1.790; CI = 1.167–2.745).

**Table 4:** Independent associations between patients’ characteristics, treatment, and the probability to be readmitted for recurrent epistaxis (patients without versus with readmission for recurrent epistaxis)

Measure		OR*	95% CI* lower	95% CI* upper	*p*
*Model 1: patients’ characteristics*					
Gender	Female	1			
	Male	1.756	1.155	2.668	**0.008**
Non-VKA oral anticoagulant (NOAC)	No	1			
	Yes	1.731	1.046	2.865	**0.033**
Anticoagulant combination therapy	0 or 1 drug	1			
	2 or 3 drugs	1.484	0.767	2.870	0.241
Length of stay	1–3 days	1			
	≥ 4 days	1.272	0.858	1.886	0.230
Hereditary hemorrhagic telangiectasia	No	1			
	Yes	13.216	5.102	34.231	**< 0.001**
Recurrent bleeding during inpatient treatment	No	1			
	Yes	1.790	1.167	2.745	0.008
*Model 2: treatment*					
Nose packing	No				
	Yes	1.469	0.996	2.165	0.052

Multivariable binary logistic regression for the dichotomized outcome parameter readmission for recurrent epistaxis with OR related to no readmission. Significant *p* values (*p* < 0.05) in bold

*OR* odds ratio, *CI* confidence interval

## Discussion

The presented population-based study on an actual and complete year of inpatient treatment for severe epistaxis in a territorial federal state in Germany revealed that the affected population was mainly elderly and comorbid persons with male predominance. The incidence was strongly increases beyond 70 years of age. Beyond nasal packing, treatment decisions were variable. Half of the patients needed a length of stay of ≥ 4 days. Anti-platelet drug use, need to pause of anticoagulation therapy, localization of the bleeding not in the anterior region, recurrent bleeding during inpatient treatment were patient factors leading to longer inpatient length of stay. The stay was also longer if electrocoagulation is not possible to stop the nose bleeding, or use of blood transfusion. Male gender, oral anticoagulant use, hereditary hemorrhagic telangiectasia, and recurrent bleeding during index inpatient treatment were important risk factors for later readmission because of recurrent epistaxis. Decision making for inpatient treatment of epistaxis should be standardized by the use of clinical guidelines to implement strategies to shorten inpatient treatment and to reduce the risk of readmission.

There are only a few actual population-based studies on inpatient treatment of epistaxis [2, 3, 6, 14]. Furthermore, there is only one other population-based study on factors influencing readmission for recurrent epistaxis [8]. A strength of the present study is the combination of population-based data (all epistaxis inpatients of one federal state in one year) with hospital-based data as the hospital charts of all 840 patients were additionally analyzed. Therefore, detailed data on patients’ characteristics, diagnostic results, and treatment in daily practice beyond clinical trials could be investigated.

The retrospective design is a limitation of the study. A selection bias cannot be ruled out as the criteria to admit the patients for inpatient treatment remain unclear. Furthermore, several important parameters could not be assessed. Epistaxis is an important mortality factor but mortality could not be estimated [3, 15]. A low socio-economic status and deprivation have been shown as reason for admission, too, but could not be investigated [3].

The incidence of ENT/emergency department visits was estimated with 108/100,000 population per year for the United Kingdom and with about 170/100,000 (1200/100,000 for patients aged 70–79 years) for the United States in the last decade [2, 6]. About 5% of these patients required admission [6]. The admission policies might be different, but compared to these studies, the number of patients needing admission for epistaxis was much higher in Thuringia a century later. This trend can be seen all over Germany [16]. The strong increase of incidence in elderly people that was seen already a century ago might have risen further [2].

Others have also shown a male predominance, cardiovascular disease as frequent comorbidity, and overpresentation of patients with anti-platelet drug and anticoagulant use [3, 5, 7, 8, 17, 18]. Data on duration of admission are sparse. The main duration of inpatient treatment in Wales from 1995 to 2009 was 3.2 days [7]. We could not identify any other study analyzing risk factors for a longer duration of stay. Epistaxis patients have a high risk of about 14–20% of readmission with recurrent epistaxis [8, 19]. This could be confirmed by the present study. Recently, Chaaban et al. revealed by multivariate analysis that age > 75 years, male gender, anterior packing/cautery, congestive heart failure, diabetes mellitus, and obstructive sleep apnea were independent predictors for readmission in a series of 4120 patients (5% of the United States sample of Medicare data from 2012 to 2013) [8]. Cohen et al. further differentiated between early (within 30 days) and late readmission [19]. Based on multivariate analysis in a case series of 653 patients treated in a single Israeli academic center, prior nasal surgery and anemia were independent risk factors for early readmission.

First, it would be helpful to establish a standardized clinical guideline for epistaxis management at index presentation, decision making for inpatient treatment, and standardized inpatient treatment. This might help to reduce admission rates and length of hospital stay [20]. Of course, nasal packing might be the only first choice of treatment in severe epistaxis, but it leads to longer hospital stay and is a risk factor for readmission [21]. Especially for anterior epistaxis, identification of the bleeding source and treatment by electrocautery or chemical cautery increases the success rate and reduces the risk of recurrent bleeding [21]. Therefore, early removal of the nasal packing in such patients (after initial stabilization, blood pressure regulation, and stop of anticoagulants) might be worthwhile to repeat a careful endoscopy of the nose, identify and coagulate the bleeding source. In patients with continuous posterior bleeding, early indication for endoscopic sphenopalatine artery cauterization might also help to reduce the risk of recurrent bleeding [22].

## Conclusions

Male gender, oral anticoagulant use, hereditary hemorrhagic telangiectasia, and recurrent bleeding during index inpatient treatment were the important risk factors for readmission. A strategy to be analyzed in further studies might be (a) to arrange controls at the general physician in regard of, for instance, anticoagulant use, blood pressure control, or nasal mucosa care in patients with hereditary hemorrhagic telangiectasia, or (b) to schedule strict follow-up visits for these patients at risk to see if this strategy helps to reduce the risk for recurrent nose bleeding.

## Electronic supplementary material

Below is the link to the electronic supplementary material.

Supplementary file1 (DOCX 56 kb)
