# Supplementary material for: Hospitalization for epistaxis: a population-based healthcare research study in Thuringia, Germany
Source: Eur Arch Otorhinolaryngol. 2020 Mar 2;277(6):1659–66. doi: 10.1007/s00405-020-05875-2 (PMC7198635; doi:10.1007/s00405-020-05875-2)
Supplement: Supplementary file 1 — Supplementary file1 (DOCX 56 kb) [file 405_2020_5875_MOESM1_ESM.docx]

**Supplementary File**

**Supplement Table 1**

| **Supplement Table 1.** Blood coagulation and blood pressure at admission. | | |
| --- | --- | --- |
| **Parameter** | **N** | **%** |
| International normalized ratio |  |  |
| <0,99 | 196 | 23.3 |
| 1.00-1.49 | 363 | 43.2 |
| 1.50-1.99 | 59 | 7.0 |
| 2.00-2.49 | 63 | 7.5 |
| 2.50-2.99 | 34 | 4.0 |
| 3.00-3.49 | 25 | 3.0 |
| 3.50-3.99 | 11 | 1.3 |
| 4.00-4.49 | 5 | 0.6 |
| 4.50-4.99 | 6 | 0.7 |
| >5,00 | 9 | 1.1 |
| Missing | 68 | 8.1 |
| Quick value |  |  |
| Normal (≥70%) | 501 | 59.6 |
| Low (<70%) | 271 | 32.3 |
| Blood pressure |  |  |
| Normal | 162 | 19.3 |
| Hypotonic | 66 | 7.9 |
| Hypertonic | 417 | 49.6 |
| Hypertensive crisis | 107 | 12.7 |
| Missing | 88 | 10.5 |
|  | **Mean±SD** | **Median, range** |
| Blood pressure, systolic, mmHg | 145.6±28.0 | 140.0, 55-250 |
| Blood pressure, diastolic, mmHg | 83.2±15.6 | 80.0, 45-160 |
| Thrombocytes, Gpt/l | 231.2±82.4 | 226.5, 4-701 |
| Partial thromboplastin time (PTT), sec | 34.6±10.5 | 32.1, 17-153.0 |
| Mean corpuscular hemoglobin (MCH), fmol | 1.8±0.3 | 1.9, 0.9-2.4 |
| Mean corpuscular hemoglobin concentration (MCHC), mmol/l | 20.6±0.9 | 20.6, 15.8-22.7 |
| Hemoglobin, mmol/l | 7.9±1.4 | 8.1, 2.8-12.6 |

SD, standard deviation

**Supplement Table 2**

| **Supplement Table 2**. Incidence per 100.000 population of epistaxis inpatient treatments in Thuringia in 2016 of the patients living in Thuringia (N=763; 90.8% of the study population). | | | | | |
| --- | --- | --- | --- | --- | --- |
| Age, years | All | Men | Women | Male:female ratio |  |
| **≤5** | 22 | 26 | 18 | 1:0.69 |  |
| **5≤10** | 13 | 15 | 12 | 1:0.80 |  |
| **10≤15** | 9 | 9 | 10 | 1:1.11 |  |
| **15-20** | 12 | 18 | 5 | 1:0.28 |  |
| **20≤25** | 6 | 12 | 0 | 1:0.00 |  |
| **25≤30** | 7 | 9 | 5 | 1:0.56 |  |
| **30≤35** | 7 | 4 | 11 | 1:2.75 |  |
| **34≤40** | 8 | 10 | 5 | 1:0.50 |  |
| **40≤45** | 6 | 6 | 5 | 1:0.83 |  |
| **45≤50** | 14 | 23 | 4 | 1:0.17 |  |
| **50≤55** | 18 | 26 | 11 | 1:0.42 |  |
| **55≤60** | 25 | 37 | 13 | 1:0.35 |  |
| **60≤65** | 45 | 58 | 33 | 1:0.57 |  |
| **54≤70** | 37 | 48 | 26 | 1:0.54 |  |
| **70≤75** | 81 | 112 | 55 | 1:0.49 |  |
| **75≤80** | 109 | 135 | 89 | 1:0.66 |  |
| **80≤85** | 130 | 182 | 97 | 1:0.53 |  |
| **>85** | 140 | 222 | 108 | 1:0.49 |  |
| **All** | 35 | 42 | 28 | 1:0.67 |  |

**Supplement Table 3**

| **Supplement Table 3.** Comparison of patients with short (1-3 days) versus with longer (≥4 days) of length of inpatient stay. | | | |
| --- | --- | --- | --- |
| **Parameter** | **Length of stay**  **1-3 days**  **N= 415** | **Length of stay**  **≥4 days**  **N=425** | **p** |
| Gender |  |  | 0.439 |
| Male | 244 | 261 |  |
| Female | 171 | 164 |  |
| Under anticoagulation therapy |  |  | 0.490 |
| Yes | 260 | 276 |  |
| No | 155 | 149 |  |
| Anti-platelet drug |  |  | **0.025** |
| Yes | 123 | 157 |  |
| No | 292 | 268 |  |
| Vitamin K antagonist (VKA) |  |  | 0.748 |
| Yes | 88 | 94 |  |
| No | 327 | 331 |  |
| Non-VKA oral anticoagulant (NOAC) |  |  | 0.194 |
| Yes | 70 | 58 |  |
| No | 345 | 367 |  |
| Anticoagulant combination therapy |  |  | **0.044** |
| No or 1 anticoagulant | 392 | 386 |  |
| 2-3 anticoagulant | 23 | 39 |  |
| Pause of anticoagulation |  |  | **0.073** |
| Yes or no anticoagulant therapy | 195 | 226 |  |
| No pause of anticoagulation | 220 | 199 |  |
| Hypertension, arterial |  |  | **0.009** |
| Yes | 221 | 264 |  |
| No | 194 | 161 |  |
| Diabetes mellitus |  |  | **0.012** |
| Yes | 80 | 113 |  |
| No | 335 | 312 |  |
| Hereditary hemorrhagic telangiectasia |  |  | 0.542 |
| Yes | 9 | 12 |  |
| No | 406 | 413 |  |
| Malignant tumor |  |  | 0.348 |
| Yes | 43 | 36 |  |
| No | 372 | 389 |  |
| Localization of the bleeding |  |  | **<0.001** |
| Anterior | 266 | 195 |  |
| Not anterior | 149 | 230 |  |
| Recurrent bleeding during inpatient treatment |  |  | **<0.001** |
| Yes | 66 | 118 |  |
| No | 349 | 307 |  |
| Hypertensive crisis, at admission* |  |  | 0.777 |
| Yes | 56 | 66 |  |
| No | 298 | 332 |  |
| Hypertension, at admission* |  |  | 0.958 |
| Yes | 247 | 277 |  |
| No | 107 | 121 |  |
| Initial Quick value* |  |  | 0.944 |
| $\geq$70% | 239 | 262 |  |
| <70% | 130 | 141 |  |
| Initial INR value* |  |  | 0.899 |
| $\leq$2 | 296 | 321 |  |
| >2 | 73 | 81 |  |
| Nose packing |  |  | **<0.001** |
| Yes | 255 | 337 |  |
| No | 160 | 88 |  |
| Electrocoagulation |  |  | **<0.001** |
| Yes | 213 | 132 |  |
| No | 202 | 293 |  |
| Cauterization, chemical |  |  | 0.246 |
| Yes | 13 | 8 |  |
| No | 402 | 417 |  |
| Blood transfusion |  |  | **0.005** |
| Yes | 12 | 28 |  |
| No | 356 | 317 |  |
|  | **Mean±SD** | **Mean±SD** |  |
| Age, years | 64.6±24.1 | 68.4±17.2 | 0.601 |

Parameter with p-values <0.1 to be included in the multivariate analyses in bold.

**Supplement Table 4**

| **Supplement Table 4.** Comparison of patients without versus with readmission for recurrent epistaxis. | | | |
| --- | --- | --- | --- |
| **Parameter** | **No readmission**  **N=706** | **≥1 readmission**  **N=134** | **p** |
| Gender |  |  | **0.005** |
| Male | 410 | 95 |  |
| Female | 296 | 39 |  |
| Under anticoagulation therapy |  |  | 0.281 |
| Yes | 445 | 91 |  |
| No | 261 | 43 |  |
| Anti-platelet drug |  |  | 0.505 |
| Yes | 232 | 48 |  |
| No | 474 | 86 |  |
| Vitamin K antagonist (VKA) |  |  | 0.488 |
| Yes | 156 | 26 |  |
| No | 550 | 108 |  |
| Non-VKA oral anticoagulant (NOAC) |  |  | **0.047** |
| Yes | 100 | 28 |  |
| No | 606 | 106 |  |
| Anticoagulant combination therapy |  |  | **0.066** |
| No or 1 anticoagulant | 659 | 119 |  |
| 2-3 anticoagulant | 47 | 15 |  |
| Pause of anticoagulation |  |  | 0.827 |
| Yes or no anticoagulant therapy | 355 | 66 |  |
| No pause of anticoagulation | 351 | 68 |  |
| Length of stay |  |  | **0.083** |
| 1-3 days | 358 | 57 |  |
| ≥4 days | 348 | 77 |  |
| Hypertension, arterial |  |  | 0.377 |
| Yes | 403 | 82 |  |
| No | 303 | 52 |  |
| Diabetes mellitus |  |  | 0.472 |
| Yes | 159 | 34 |  |
| No | 547 | 100 |  |
| Hereditary hemorrhagic telangiectasia |  |  | **<0.001** |
| Yes | 7 | 14 |  |
| No | 699 | 120 |  |
| Malignant tumor |  |  | 0.652 |
| Yes | 65 | 14 |  |
| No | 641 | 120 |  |
| Localization of the bleeding |  |  | 0.782 |
| Anterior | 386 | 75 |  |
| Not anterior | 320 | 59 |  |
| Recurrent bleeding during inpatient treatment |  |  | **0.002** |
| Yes | 141 | 43 |  |
| No | 565 | 91 |  |
| Hypertensive crisis, at admission* |  |  | 0.171 |
| Yes | 98 | 24 |  |
| No | 537 | 93 |  |
| Hypertension, at admission* |  |  | 0.153 |
| Yes | 449 | 75 |  |
| No | 186 | 42 |  |
| Initial Quick value* |  |  | 0.971 |
| $\geq$70% | 421 | 88 |  |
| <70% | 228 | 43 |  |
| Initial INR value* |  |  | 0.261 |
| $\leq$2 | 514 | 103 |  |
| >2 | 134 | 20 |  |
| Nose packing |  |  | **0.051** |
| Yes | 507 | 85 |  |
| No | 199 | 49 |  |
| Electrocoagulation |  |  | 0.178 |
| Yes | 297 | 48 |  |
| No | 409 | 86 |  |
| Cauterization, chemical |  |  | 0.319 |
| Yes | 16 | 5 |  |
| No | 690 | 129 |  |
| Blood transfusion |  |  | 0.766 |
| Yes | 32 | 8 |  |
| No | 551 | 122 |  |
|  | **Mean±SD** | **Mean±SD** |  |
| Age, years | 65.9±21.6 | 69.8±16.8 | 0.154 |
| Duration of inpatient treatment, days | 4.1±3.2 | 4.7±2.9 | **0.002** |

*values missing for some patients; parameter with p-values <0.1 to be included in the multivariate analyses in bold.
